# Platelet-derived exosomes alleviate tendon stem/progenitor cell senescence and ferroptosis by regulating AMPK/Nrf2/GPX4 signaling and improve tendon-bone junction regeneration in rats

**DOI:** 10.1186/s13018-024-04869-8

**Published:** 2024-06-28

**Authors:** Deheng Chen, Qian Tang, Wei Song, Yaohua He

**Affiliations:** 1 Department of Orthopedics, Shanghai Sixth People’s Hospital Affiliated to Shanghai Jiao Tong University of Medicine, 600 Yishan Road, Shanghai, 200233 China; 2 Department of Orthopedics, Jinshan Branch of Shanghai Sixth People’s Hospital Affiliated to Shanghai University of Medicine & Health Sciences, 147 Jiankang Road, Shanghai, 201503 China

**Keywords:** Platelet-derived exosomes, Senescence, Ferroptosis, AMPK/Nrf2/GPX4

## Abstract

**Background:**

Tendon stem/progenitor cell (TSPC) senescence contributes to tendon degeneration and impaired tendon repair, resulting in age-related tendon disorders. Ferroptosis, a unique iron-dependent form of programmed cell death, might participate in the process of senescence. However, whether ferroptosis plays a role in TSPC senescence and tendon regeneration remains unclear. Recent studies reported that Platelet-derived exosomes (PL-Exos) might provide significant advantages in musculoskeletal regeneration and inflammation regulation. The effects and mechanism of PL-Exos on TSPC senescence and tendon regeneration are worthy of further study.

**Methods:**

Herein, we examined the role of ferroptosis in the pathogenesis of TSPC senescence. PL-Exos were isolated and determined by TEM, particle size analysis, western blot and mass spectrometry identification. We investigated the function and underlying mechanisms of PL-Exos in TSPC senescence and ferroptosis via western blot, real-time quantitative polymerase chain reaction, and immunofluorescence analysis in vitro. Tendon regeneration was evaluated by HE staining, Safranin-O staining, and biomechanical tests in a rotator cuff tear model in rats.

**Results:**

We discovered that ferroptosis was involved in senescent TSPCs. Furthermore, PL-Exos mitigated the aging phenotypes and ferroptosis of TSPCs induced by t-BHP and preserved their proliferation and tenogenic capacity. The in vivo animal results indicated that PL-Exos improved tendon-bone healing properties and mechanical strength. Mechanistically, PL-Exos activated AMPK phosphorylation and the downstream nuclear factor erythroid 2-related factor 2 (Nrf2)/glutathione peroxidase 4 (GPX4) signaling pathway, leading to the suppression of lipid peroxidation. AMPK inhibition or GPX4 inhibition blocked the protective effect of PL-Exos against t-BHP-induced ferroptosis and senescence.

**Conclusion:**

In conclusion, ferroptosis might play a crucial role in TSPC aging. AMPK/Nrf2/GPX4 activation by PL-Exos was found to inhibit ferroptosis, consequently leading to the suppression of senescence in TSPCs. Our results provided new theoretical evidence for the potential application of PL-Exos to restrain tendon degeneration and promote tendon regeneration.

## Introduction

Degenerative rotator cuff tendon injuries/tears are among the most common musculoskeletal disorders and often occur in elderly individuals [1]. Tendon stem/progenitor cells (TSPCs) serve as the primary intrinsic origin of tenocytes, fulfilling crucial functions in maintaining tendon homeostasis and regeneration. However, senescence can cause changes in TSPC characteristics. Senescent TSPCs exhibit diminished self-renewal and proliferation with maintenance of multipotency, inducing tendon degeneration and regeneration impairment [2]. Although it is commonly recognized that senescent TSPCs undergo a decline in functionality, further investigation is necessary to develop more effective and precise techniques for rejuvenating the function of aged TSPCs and restoring their impaired capacity to repair tendon injury.

Ferroptosis is a nonapoptotic form of cell death that occurs in an iron-dependent and reactive oxygen species (ROS)-dependent manner [3, 4]. Ferroptosis is involved in the pathological mechanisms of cardiovascular disease, neurodegeneration, and other age-related diseases [5]. Recent researches have shown that suppressing ferroptosis by the inhibition of lipid peroxidation or the restriction of iron retention may decrease age-related cell death and prolong the lifespan and health span of the nematode [6]. Iron accumulation in tissues is often observed to be greater in older individuals. Iron overload can induce ferroptosis by generating reactive oxygen species (ROS) through the Fenton reaction [7]. Increased oxidative stress is considered a major cause of aging. Thus, the involvement of ferroptosis in the aging process is reasonable. However, whether ferroptosis plays a role in TSPCS senescence and tendon regeneration remains unclear.

Nuclear factor erythroid 2-related factor 2 (NRF2) is a transcription factor pivotal for regulating antioxidant stress and inflammatory responses. Nrf2 was recently reported to play a critical role in mitigating lipid peroxidation and ferroptosis by regulating the downstream signal GPX4 and iron homeostasis [8]. AMP-activated protein kinase (AMPK) has been demonstrated to function as a resilient node for sensing nutrient-related signals associated with the aging process [9]. Previous studies demonstrated that activation of AMPK modulates stem cell senescence [10]. A recent study revealed that AMPK activation inhibited ferroptosis by inactivating acetyl-CoA carboxylase (ACC) and restraining the biosynthesis of polyunsaturated fatty acids [11]. Furthermore, AMPK phosphorylation also promotes the nuclear translocation and activation of Nrf2 [12]. Currently, there is a lack of research on the potential mechanism by which the AMPK/Nrf2 pathway regulates ferroptosis and senescence in TSPCs.

Platelets are the initial responders to bodily injury and have demonstrated an impressive capacity to stimulate tissue regeneration and healing [13]. Platelets and their derivatives, such as platelet-rich plasma (PRP) and platelet lysate(PL), are often used in tissue regeneration. However, the absence of appropriate standardization has resulted in a wide variety of products with varying biological and therapeutic effects [14]. Platelet-rich plasma consists of abundant growth factors, cytokines and immunomodulators produced by platelets. These factors may be released as free proteins or inside exosomes in a more concentrated form than in the parent cell [15].Platelet-derived exosomes (PL-Exos) are membrane vesicles produced by platelets upon activation. PL-Exos have been widely used in the field of regenerative medicine, including for wound healing, neuroregeneration, and bone repair [16, 17]. A recent study also showed that exosomes from young human plasma facilitated functional recovery after intracerebral hemorrhage by mitigating ferroptotic damage [18]. However, the effect and mechanism of PL-Exos treatment on tendon regeneration remain unclear. This study aimed to determine whether the senescence phenotype is linked to ferroptosis in TSPCs. Moreover, we elucidated the effect and mechanism of PL-Exos on TSPC senescence and tendon regeneration.

## Materials and methods

### Separation of platelet-derived exosomes

Platelet lysate (PL) was purchased from Stemery BIO-TECH Co., Ltd. (China). The exosome separation methodology was derived from a prior investigation with some modifications [19]. Subsequently, the PL was thinned with PBS and subjected to filtration (0.22 μm) for purification. It was then centrifuged at 10,000 ×g for 30 min to eliminate cell debris and medium-sized vesicles. The supernatant was subjected to ultracentrifugation at 110,000 × g for 75 min. The samples containing exosomes were subjected to two washes with phosphate-buffered saline (PBS) and ultracentrifugation at a speed of 110,000 × g for 75 min. Then, the PL-Exos were suspended in 500 µl of PBS.

### TSPC isolation and culture

TSPCs were acquired following the methodology outlined in a prior study [20]. Achilles tendons were collected from Sprague‒Dawley rats (male, 8-week-old and 80-week-old). The tendons that were collected were divided into sections and subjected to digestion using type I collagenase at a concentration of 3% (Sigma‒Aldrich). The tendon tissues were cultured in Dulbecco’s modified Eagle’s medium (Gibco) supplemented with 12% fetal bovine serum (Gibco) and 1% penicillin‒streptomycin. The nucleated cells were plated at a low cell density of 50 nucleated cells/cm^2^ to isolate the stem cells. After 7 days, the cells were trypsinized and then merged to create passage 0. Experiments were conducted using cells from P2 to P6. The ability of the cells to form colonies and differentiate into multiple lineages was verified prior to their use in the experiments using established assays described in a previous study [20].

### Characterization of the PL-Exos

Nanoparticle tracking analysis was utilized to evaluate the dimensions and density of the PL-Exos (NanoFCM, China). The morphology of the PL-Exos was assessed via transmission electron microscopy (Thermo Fisher, USA). The purity and characteristics were analyzed via Western blot. The expression of the exosome markers CD63, CD9 and calnexin was examined.

### Proteomic analysis

Platelet-derived exosomes were collected and digested using protein lysis buffer. The protein mixtures were then isolated, processed, and digested according to the standard operating procedure developed by Biotree Biotech. The peptide mixtures were examined using an Orbitrap Fusion mass spectrometer with a nanoflow liquid chromatography system. The vendor’s raw MS files were analyzed using Proteome Discoverer (PD) software (version 2.4.0.305) and the integrated Sequest HT search engine. The raw mass spectrometry data have been submitted to the ProteomeXchange Consortium via the iProX partner repository. The dataset was authenticated using the PXD number PXD045219.

### Western blot analysis

A mixture of RIPA buffer and protease and phosphatase inhibitors was used to lyse the cells. The protein concentrations were determined using a BCA kit (Beyotime Biotechnology). Protein isolation was performed using 10% SDS‒PAGE, and proteins were subsequently transferred to PVDF membranes. The membranes were incubated with 5% skim milk powder for 2 h, followed by overnight incubation at 4 °C with the following primary antibodies: anti-CD63 (Abcam, 1:1500); anti-CD9 (Abcam, 1:1000); anti-Calnexin (Abcam, 1:1000); anti-p-AMPK (CST, 1:500); anti-GPX4 (Abcam, 1:1000); anti-Nrf2 (Abcam, 1:500); anti-PCNA (Abcam, 1:2000); anti-ACSL4 (Abcam, 1:500); anti-p-p53 (Abcam, 1:1000); anti-p16^INK4a^ (Santa Cruz, 1:1000); anti-GAPDH (CST, 1:2500). Next, the membranes were incubated with the appropriate secondary antibody for 90 min and exposed (Bio-Rad).

### RT-qPCR

Total RNA was isolated by TRIzol-up reagent (EZBioscience). Reverse transcription and real-time PCR experiments were conducted using an Applied Biosystems 7500 Real-Time PCR system following the manufacturer’s instructions. For gene expression analysis, the 2^(-ΔΔCt) method was used to calculate the relative expression level of the target gene, which was then normalized to that of GAPDH. The primers used for PCR are listed in Table 1.

**Table 1:** Primer sequences for RT-qPCR

Target gene	Forward	Reverse
MMP3	5′-CATGAACTTGGCCACTCCCT-3′	5′-TGGGTACCACGAGGACATCA-3′
IL6	5′-AAGAGACTTCCAGCCAGTTGCC-3′	5′-TGTGGGTGGTATCCTCTGTGAAG-3′
IL1B	5′-TGACCTGTTCTTTGAGGCTGAC-3′	5′-CATCATCCCACGAGTCACAGAG-3′
CXCL5	5′-ATTCACCCTGCTGGCATTTCT-3′	5′-GCTTGTGGGTCAAGACAAACAT-3′
Scx	5′ -CGAGAACACCCAGCCCAAAC-3′	5′-CGTCTTTCTGTCACGGTCTTTG-3
TNMD	5′-GACCTATGGCATGGAGCACAC-3′	5′- TGTTTCATCGGTGCCATTTCC-3′
MKX	5′-AAGGTGAGGCACAAGCGACA-3′	5′-ACTAGCGTCATCTGCGAGCCT-3′
COL1A1	5′-AGAGGCATAAAGGGTCATCGTG-3′	5′-AGACCGTTGAGTCCATCTTTGC-3′
PTGS2	5′- ACG​TGT​TGA​CGT​CCA​GAT​CA -3′	5′- ACGTGGGGAGGGTAGATCAT − 3′
SLC7A11	5′- GTGTTTGCTGTCTCCAGGTTAT − 3′	5′- TCTTTAGAGTCTTCTGGTACAACTT − 3′
GAPDH	5′-AGGTCGGTGTGAACGGATTTG-3′	5′-TGTAGACCATGTAGTTGAGGTCA-3′

### β-galactosidase staining

The β-galactosidase (β-gal) assay was conducted utilizing an SA-β-gal staining kit (Beyotime). The proportion of senescent cells was determined by dividing the count of positive cells by the total cell count in a specific area. A minimum of 300 cells were enumerated in every group.

### Immunofluorescence staining

The cells were fixated with paraformaldehyde for 15 min before being blocked with 5% BSA for 40 min. Primary antibodies against GPX4 (Abcam, 1:200), Col I (Abcam, 1:500), Nrf2 (Abcam, 1:150), p21 (Abcam, 1:200) and 4-HNE (Abcam, 1:100) were applied to the cells, which were then incubated overnight at 4 °C. Finally, FITC- or CY5-conjugated secondary antibodies were added and incubated for 90 min. The nuclei were stained with DAPI and observed using a fluorescence microscope (Carl Zeiss).

### Assessment of lipid peroxidation and the levels of GSH and mtROS

The lipid peroxidation levels and GSH levels of TSPCs or tendon tissues were examined using a Lipid Peroxidation (MDA) Assay Kit (ab233471, Abcam), and Glutathione Assay Kit (MAK364, Sigma‒Aldrich), respectively, following the manufacturer’s instructions.

The levels of mitochondrial reactive oxygen species (mtROS) in TSPCs were measured via MitoSOX probes from Thermo Fisher, USA. TSPCs on slides were treated and then exposed to MitoSOX (5 µM) for 10 min in the absence of light. Cell nuclei were labeled with DAPI. The mtROS levels in TSPCs were determined via confocal microscopy.

### Transmission electron microscopy

TSPCs were collected and fixed with 2.5% phosphateglutaraldehyde for 6 h. After postfixation, polymerization, embedding in Epon-Araldite resin and sectioning, the samples were detected with a transmission electron microscope (Thermo Fisher).

### Animal surgery

Forty-eight male Sprague‒Dawley rats (6 months old) underwent rotator cuff tear surgery as reported in our previous study [21]. After anesthetization with pentobarbital, an incision was made on the shoulder to reveal the supraspinatus tendon. The supraspinatus tendon was surgically removed at its attachment site on the humeral neck. A drill bit (0.5 mm) was utilized to create a bone tunnel in the attachment site. The supraspinatus tendon was incised and subsequently sutured in situ using a modified Mason–Allen stitch. Rats were subacromially injected with 20 µL (2 × 10^10^ particles) of PL-exosomes or PBS with ultrasonic guidance weekly, and euthanasia was performed at 4 and 8 weeks after the operation. Regular surveillance of the rats was conducted to guarantee their welfare, and all animals were provided with unrestricted mobility as well as standard food and water.

### IVIS Imaging

PL-Exosomes were incubated with DiR dye (5 µM, Meilunbio, China) for 30 min at room temperature, followed by centrifugation at 110,000 × g for 70 min at 4 °C to eliminate excess dye. Then, 20 µl of the PL-Exos@DIR solution was injected subacromially into the right shoulder joint capsule of each rat via ultrasonic guidance. The DiR-labeled exosome distribution was analyzed using an IVIS Spectrum (Perkin Elmer, Waltham, MA, USA) 2 days after surgery.

### Histomorphometric evaluation

Histochemical analysis was conducted on the animals at 4 and 8 weeks after the operation. The samples of tendon-humerus were subjected to standard fixation, decalcification, dehydration, and embedding in paraffin. Subsequently, they were sliced into 5 mm thick sections. The specimens were stained using an H&E kit from Beyotime and a safranin O/fast green staining kit from Sigma‒Aldrich following the provided instructions. Picrosirius red-stained sections were imaged using polarized light microscopy. The digitized images were then semiquantitatively analyzed for collagen organization and components using ImageJ software. The assessment of the repaired tendon was conducted using a revised scoring system for tendon maturation in a blinded manner [22].

### Immunostaining staining

The slides were rehydrated for IHC and IF staining, followed by blocking endogenous peroxidase activty using 3% hydrogen peroxide. The sections were then treated with 0.4% pepsin (Leagene, Beijing, China) in 5 mM HCl at 37 °C for 20 min to retrieve the antigens. 10% bovine serum albumin was used to inhibit nonspecific binding sites for 30 min at room temperature. The sections were incubated overnight at 4 °C using the primary anti-bodies: p16^INK4a^ (Santa Cruz, 1:200) and p53 (abcam, 1:200), GPX4 (abcam, 1:200), and 4-HNE (abcam,1:100). The sections were treated with an HRP-conjugated secondary antibody from Santa Cruz. For IF staining, sections were treated with fluorescent secondary antibody and observed under a fluorescence microscope.

### Biomechanical testing

A custom-designed biomechanical testing system (Instron Corp.) was used to test the biomechanical properties, as previously described^22^. Custom-designed screw grips were employed to secure the supraspinatus belly and the humerus. The specimen was initially subjected to a preload of 0.1 N and then loaded until failure at a rate of 10 mm/min. The stiffness and stress were determined by analyzing the recorded load–displacement curves.

### Statistical analysis

The study utilized GraphPad Prism software (version 7.0, USA). The data are presented as the means ± standard deviations (SD) and were obtained from 3 biological replicates and 3 technical replicates. Intergroup differences were assessed using Student’s t test or Mann-Whitney test. Multiple group comparisons were conducted using one-way ANOVA with Bonferroni post hoc analysis. A significance level of *p* < 0.05 is commonly accepted as indicating statistical significance.

## Results

### Ferroptosis occurs in senescent TSPCs

**Fig. 1:**
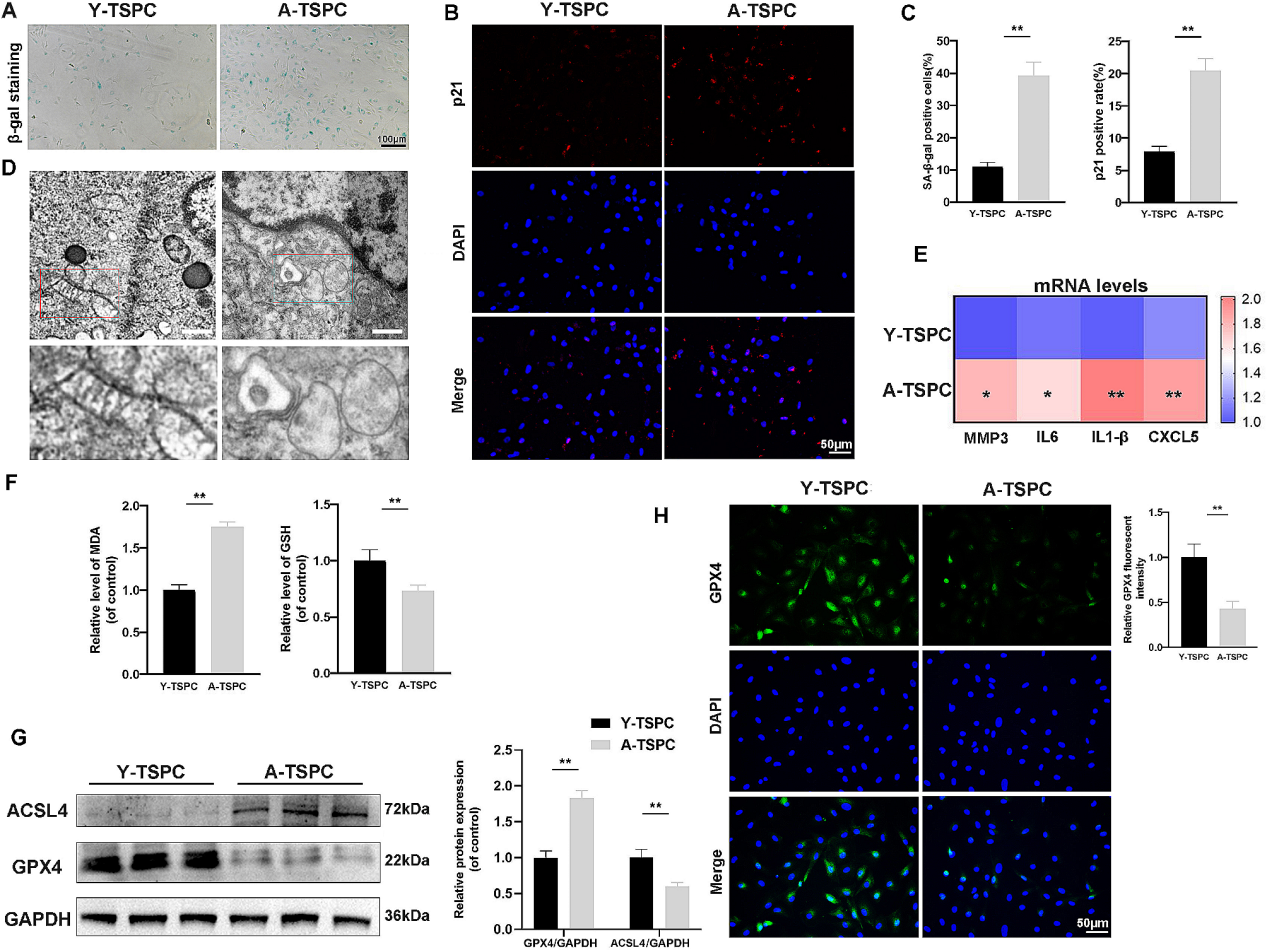
Ferroptosis is involved in senescent TSPCs. (**A**) β-gal staining of Y-TSPCs and A-TSPCs: Y-TSPCs (Young-TSPCs); A-TSPCs (Aged-TSPCs). (**B**-**C**) Immunofluorescence staining for p21. (**D**) TEM images. Scale bar: 500 nm; (**E**) RT‒qPCR analysis of specific mRNA expression levels of MMP3, IL-6, IL-1β, and CXCL5; (**F**) Relative MDA and GSH levels in TSPCs; (**G**) Western blot analysis of ferroptosis-related markers ACSL4 and GPX4; (**H**) Immunofluorescence staining for GPX4 in TSPCs. Scale bar: 50 μm. ∗*p* < 0.05, ∗∗*p* < 0.01

Young TSPCs (Y-TSPCs) and aged TSPCs (A-TSPCs) were isolated from rat Achilles tendons aged 8 weeks and 80 weeks, respectively. More β-gal-positive A-TSPCs than Y-TSPCs were detected (Fig. 1A). Immunofluorescence staining revealed a significant increase in p21 expression in the A-TSPC group (Fig. 1B). In addition, we examined the expression of senescence-associated secretory phenotype (SASP) genes, specifically MMP3, IL-6, IL-1β, and CXCL5. Our findings indicated that the expression of these SASP genes increased in A-TSPCs (Fig. 1E), which confirmed that TSPC senescence occurred.

To verify the occurrence of ferroptosis in aged TSPCs, we tested the levels of MDA (a lipid peroxidation marker) and GSH.and detected increases of MDA and a decrease of GSH in A-TSPCs (Fig. 1F). The expression of glutathione peroxidase (GPX4), a key ferroptosis protein, was also inhibited in aged TSPCs, as confirmed by western blotting (Fig. 1G) and immunofluorescence staining (Fig. 1H). Furthermore, transmission electron microscopy revealed mitochondrial vacuolization with irregular or absent cristae in aged TSPCs (Fig. 1D). In conclusion, these results indicated that ferroptosis might occur in aged TSPCs.

### Characterization and proteomic analysis of platelet-derived exosomes

**Fig. 2:**
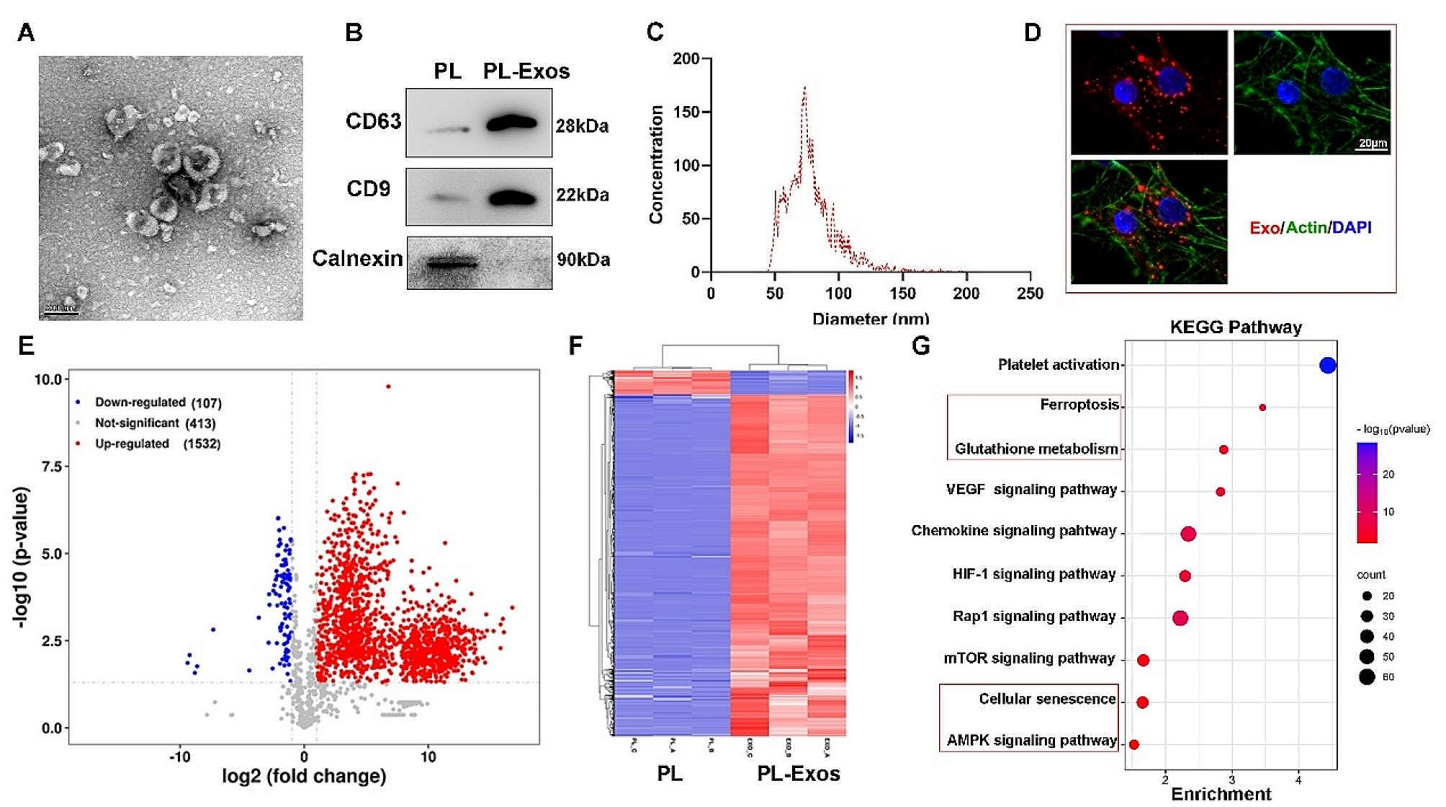
Characterization of platelet-derived exosomes. (**A**) Images of PL-Exos observed under a transmission electron microscope. Scale bar: 200 nm; (**B**) Western blot analyses of particular surface markers, including CD9, CD63 and calnexin. (**C**) NTA observation of the diameter distribution of isolated PL-Exos. (**D**) Fluorescence image of DIL-labeled exosomes (red) absorbed by TSPCs. F-actin was stained with phalloidin (green), and the nuclei were stained with DAPI (blue). Scale bar: 20 μm. (**E**) Volcano plot of differentially expressed proteins in PL-Exos versus platelet lysate; (**F**) Heatmap of the protein levels of the differentially expressed proteins; (**G**) KEGG analysis of proteins in PL-Exos compared to those in PL-Exos; (**H**) β-gal staining of TSPCs; ∗*p* < 0.05, ∗∗*p* < 0.01

The morphology of the PL-Exos was observed using TEM, revealing a saucer-like shape and bilayer membrane structure (Fig. 2A). Western blotting demonstrated that the PL-Exos exhibited enrichment of the exosomal markers CD63 and CD9 and an absence of calnexin, thereby providing additional confirmation of their identity as exosomes (Fig. 2B). Nanoparticle tracking analysis revealed that the average particle diameter of the PL-Exos was 78.3 ± 14.5 nm (Fig. 2C). PL-Exos were tracked in vitro after they were labeled with red fluorescent dye DIL and cocultured with TSPCs. Figure 2D demonstrates the efficient absorption of PL-Exos by TSPCs.

Mass spectrometry-based proteome analysis was utilized to evaluate the protein composition of the platelet-derived exosomes and platelet lysates (PL). The volcano map reveals 1639 differentially expressed proteins between PL-Exos and PL. Among these proteins, 1532 proteins were highly expressed in the PL-Exos, while 107 were downregulated (Fig. 2E). The differentially expressed proteins were then hierarchically clustered. The proteins are depicted in an expression heatmap (Fig. 2F). KEGG analysis revealed that PL-Exos were enriched in the terms platelet action, VEGF signaling and chemokine signaling. Notably, PL-Exos were found to contain proteins correlated with glutathione metabolism, ferroptosis, and the AMPK signaling pathway (Fig. 2G).

### Platelet-derived exosomes reversed TSPC senescence and degeneration

**Fig. 3:**
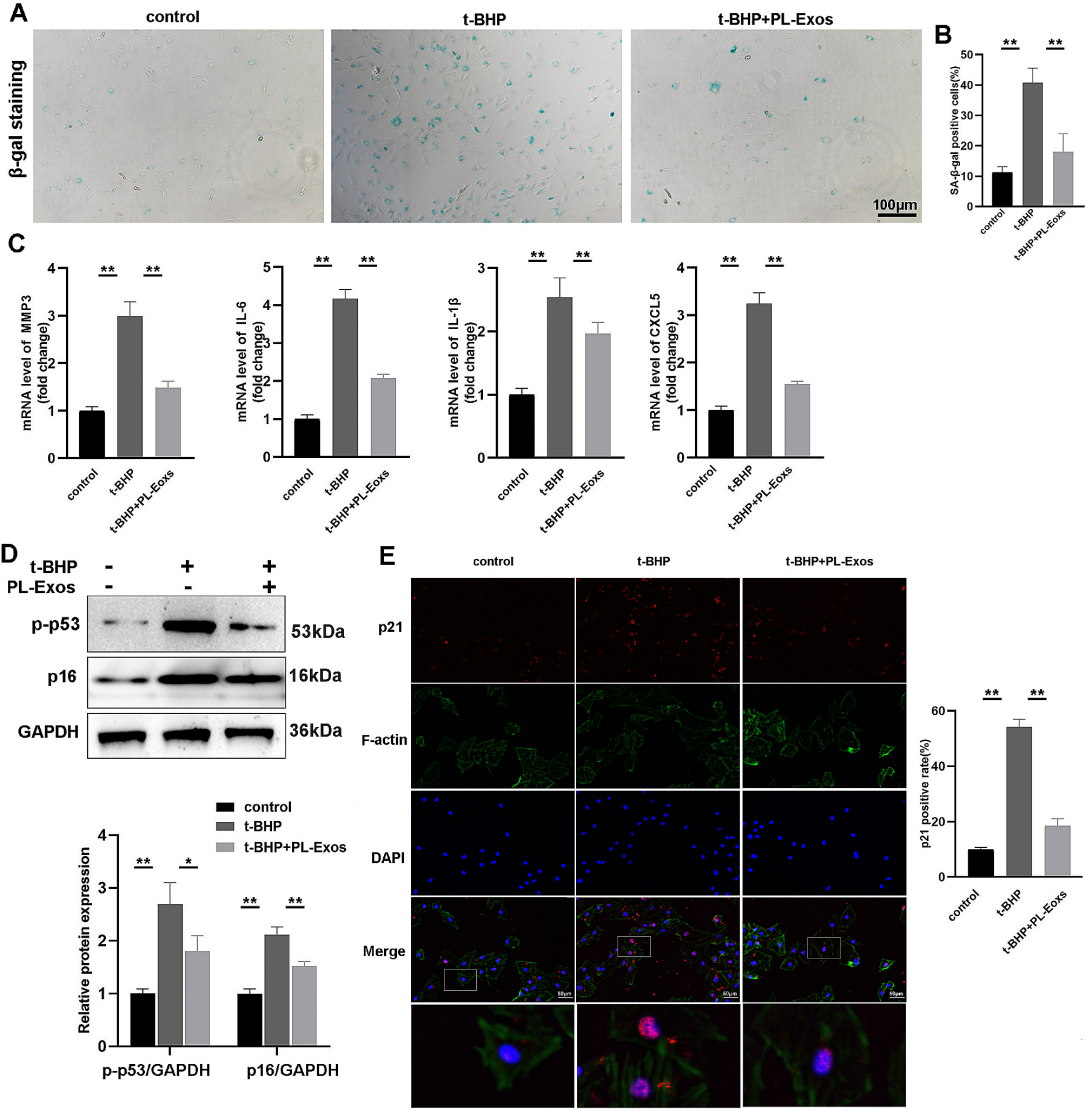
PL-Exos reverse TSPC senescence. (**A**-**B**) β-gal staining of senescent TSPCs. Scale bar: 100 μm. (**C**) RT‒qPCR analysis of the mRNA levels of SASP genes (MMP3, IL-6, IL-1β, and CXCL5). (**D**) Western blot analysis of the levels of cell cycle proteins, including p16^INK4a,^ p-p53 and GAPDH. (**E**) Immunofluorescence staining image of p21-expressing TSPCs. Scale bar = 50 μm; ∗*p* < 0.05, ∗∗*p* < 0.01

Initially, TSPCs were exposed to t-BHP (50 µM) twice a day and treated with PL-Exos (100 µg/mL). SA-β-galactosidase staining revealed an increase in the number of positive TSPCs treated with t-BHP, which was reversed by PL-Exos treatment (Fig. 3A-B). In addition, real-time PCR revealed that the expression of senescence-associated secretory phenotype SASP genes (MMP3, IL-6, IL-1β, and CXCL5) significantly increased in response to t-BHP. However, treatment with PL-Exos resulted in a reduction in SASP gene expression (Fig. 3C). Western blot analysis demonstrated that t-BHP increased the expression of cell cycle proteins, including p16^INK4a^ and p-p53 (Fig. 3D). The immunofluorescence staining of aging markers revealed a greater abundance of p21-stained TSPCs in the t-BHP-treated group, while PL-Exos considerably attenuated this alteration (Fig. 3E). Overall, PL-exosomes successfully delayed the replicative senescence and DNA damage produced by reactive oxygen species (ROS) in TSPCs.

**Fig. 4:**
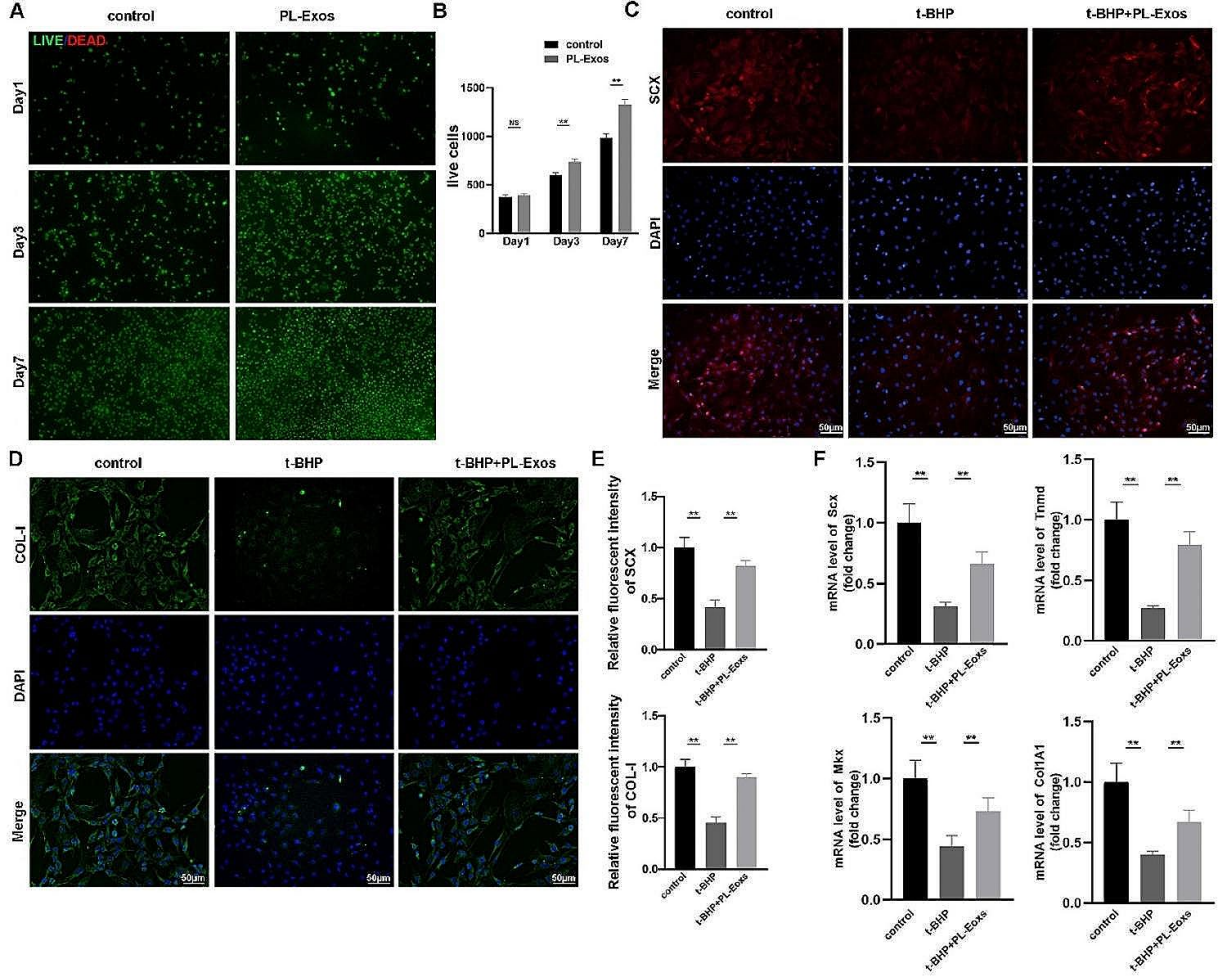
PL-Exos promote TSPC proliferation and inhibit degeneration. (**A**-**B**) Live/dead staining of TSPCs at days 1, 3, and 7. (**C**) Immunofluorescence staining image of SCX (red) and nuclei (blue). (**D**) Collagen type I was detected by immunofluorescence staining. (**E**) Quantitative analysis of SCX and collagen type I fluorescence. (**F**) mRNA expression levels of specific tendon-related genes, including scleraxis (Scx), mohawk (Mkx), tenomodulin (Tnmd), and collagen type I, in TSPCs. ∗*p* < 0.05, ∗∗*p* < 0.01

Cell viability was measured using live/dead staining, where green fluorescence represented live cells and red fluorescence represented dead cells. More live cells were observed on days 3 and 7 in the PL-Exos treated group (Fig. 4A, B), showing that PL-Exos might promote TSPC proliferation.

To investigate the effects of PL-Exos on the tenogenic differentiation capacity of cells, we analyzed the expression of the main tendon-related markers of TSPCs, namely, scleraxis (Scx), mohawk (Mkx), tenomodulin (Tnmd), and collagen type I, by quantitative real-time PCR. The expression of these genes decreased in TSPCs treated with t-BHP, while PL-Exos restored the decreased levels of tenogenic markers (Fig. 4F). The results of the immunofluorescence evaluation of SCX and collagen Type-I expression aligned with the mRNA findings. (Fig. 4C-E). These findings showed that PL-Exos effectively diminished TSPC senescence and degeneration.

### Platelet-derived exosomes hindered ferroptosis in TSPCs

**Fig. 5:**
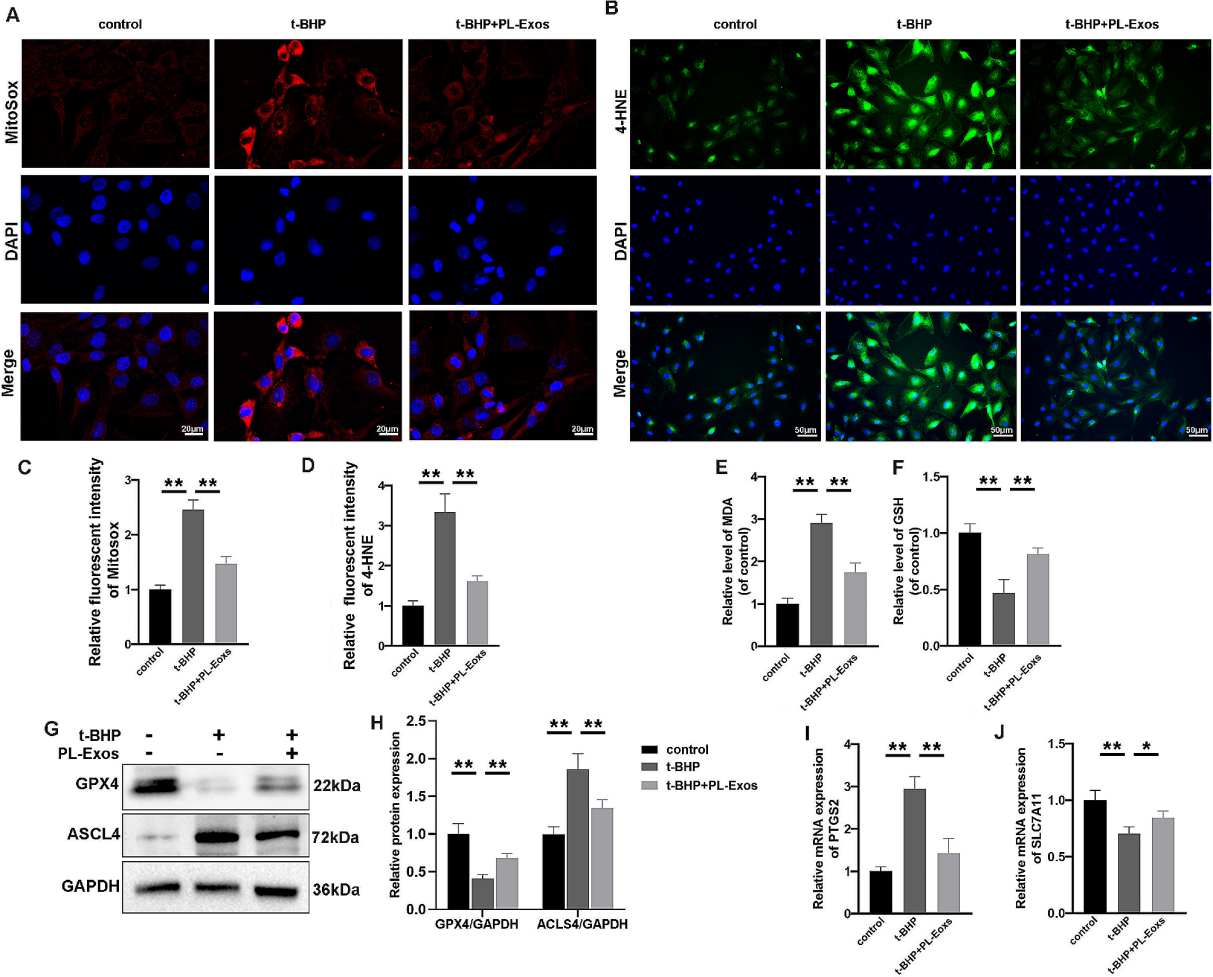
PL-Exos hindered ferroptosis in TSPCs. (**A**) Representative images showing the measurement of MitoSOX fluorescence intensity in cells. Scale bar = 20 μm. (**B**) Immunofluorescence image of 4-HNE staining. Scale bar = 50 μm; (**C**-**D**) quantification of relative fluorescence intensity; (**E**-**F**) relative levels of MDA and GSH in TSPCs; (**G**,**H**) Western blot analysis of ACSL4 and GPX4. (I, J) mRNA levels of ferroptosis-related genes (PTGS2 and SLC7A11). ∗*p* < 0.05, ∗∗*p* < 0.01

The levels of mitochondrial reactive oxygen species (ROS) were measured to determine the effects of PL-Exos and t-BHP on mitochondrial homeostasis in TSPCs. As shown in Fig. 5A, the fluorescence intensity of MitoSOX was greater in the t-BHP groups than in the control group but was lower in the PL-Exos group. As lipid peroxidation is thought to be a prominent indicator of ferroptosis, we measured the MDA and 4-HNE levels among the groups. Figure 5B shows that the fluorescence intensities of 4-HNE were greater in the t-BHP groups than in the control group but were lower in the PL-Exos group. Furthermore, our study revealed that the t-BHP-stimulated group had increased levels of MDA compared to the control group, whereas GSH levels were lower. Moreover, PL-Exos effectively mitigated the aforementioned alterations (Fig. 5E, F). Western blot analysis revealed that the expression of GPX4 decreased in the t-BHP-stimulated group compared with the control group, whereas the expression of ACSL4 increased. Additionally, the administration of PL-Exos reversed the alterations in these proteins (Fig. 5G, H). Moreover, compared with those in the control group, t-BHP exposure increased the mRNA expression of PTGS2 and decreased SLC7A11 expression in TSPCs in comparison to the control group (Fig. 4I, J). This upregulation was inhibited by the PL-Exos. The findings presented here provided compelling evidence supporting the inhibitory effect of PL-Exos on ferroptosis in TSPCs.

### The effect of PL-Exos on senescence and ferroptosis in TSPCs was mediated by the AMPK/Nrf2/GPX4 pathway

**Fig. 6:**
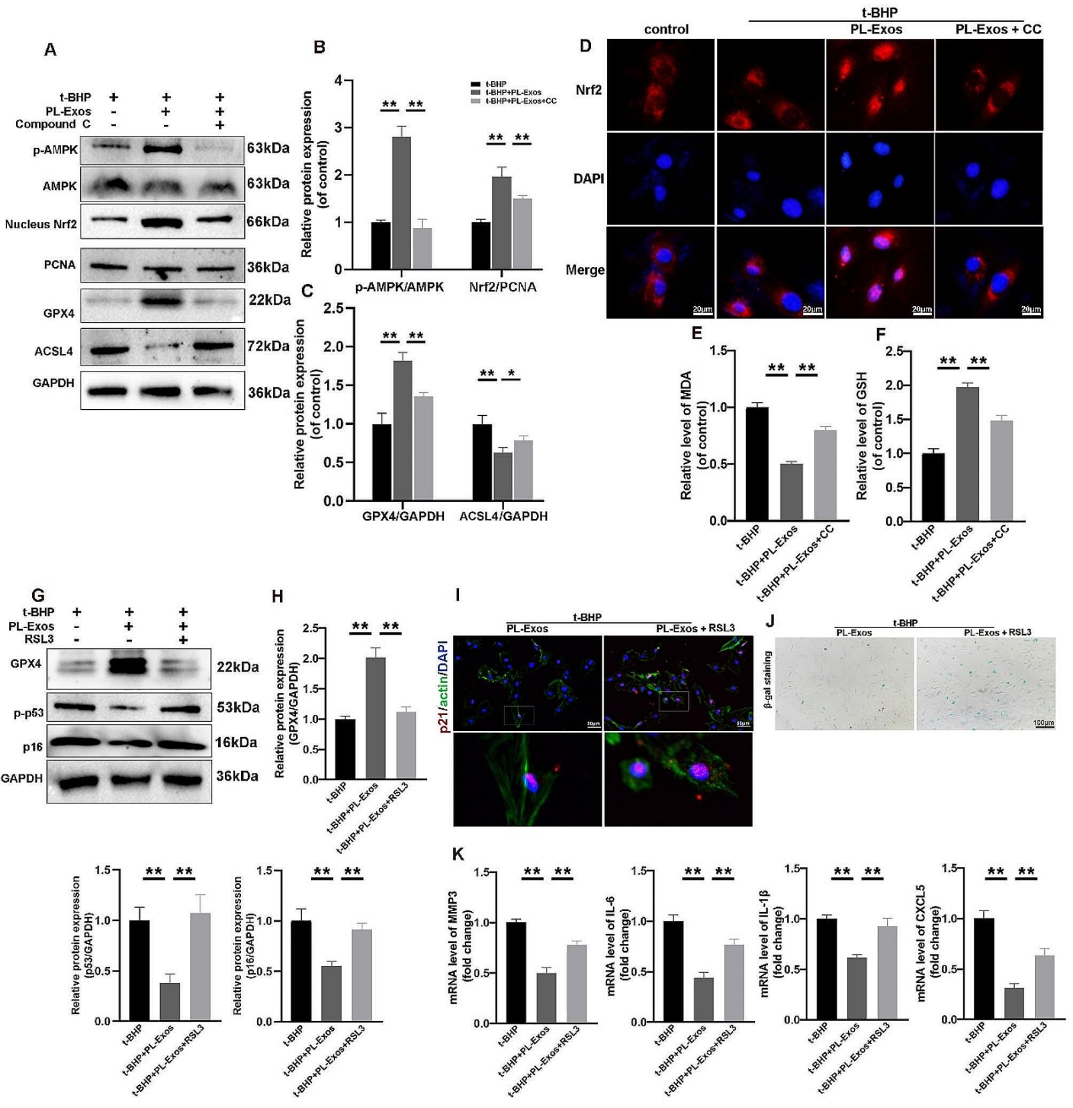
The effect of PL-Exos on senescence and ferroptosis in TSPCs is mediated by the AMPK/Nrf2/GPX4 pathway. (**A**, **B**,**C**) p-AMPK, nuclear Nrf2, PCNA, GPX4, ACSL4 and GAPDH expression was detected by Western blotting. (**D**) Immunofluorescence staining image of Nrf2 localization in TSPCs; scale bar = 20 μm. ( **E**, **F**) Relative MDA and GSH levels in TSPCs. (**G**, **H**) Western blot analysis of GPX4, p-p53, p16^INK4A^ and GAPDH expression. (**I**) Immunofluorescence staining images of p21-positive TSPCs; scale bar = 50 μm. (**J**) Representative micrographs of TSPCs stained with SA-β-gal; scale bar = 100 μm. (**K**) RT‒qPCR analysis of the mRNA levels of MMP3, IL-6, IL-1β, and CXCL5. ∗*p* < 0.05, ∗∗*p* < 0.01

To examine the involvement of AMPK/Nrf2 signaling in the protective effects of PL-Exos, TSPCs were pretreated with compound C (CC) (10 µM), a small-molecule antagonist of AMPK. As shown in Fig. 3A-B, PL-Exos enhanced the expression of phosphorylated AMPK and nuclear Nrf2, while CC reversed the PL-Exos-induced alteration of the AMPK/Nrf2 pathway (Fig. 6A-C). Immunofluorescence staining demonstrated that Nrf2 was activated and translocated into the nucleus in PL-Exos treated TSPCs, while CC abolished these effects (Fig. 6D). Notably, AMPK inhibition also negated the protective effects of PL-Exos on GPX4 upregulation and ACSL4 inhibition-mediated suppression of ferroptosis (Fig. 6A). CC treatment increased MDA levels but reduced the GSH level (Fig. 6E-F). Therefore, the protective effects of PL-Exos exerted via Nrf2 activation on t-BHP-induced ferroptosis were AMPK dependent.

To further explore the potential relationship between ferroptosis and senescence, we used RSL3 (1 µM), an inhibitor of GPX4, to induce ferroptosis. Western blot analysis revealed that RSL3 significantly inhibited GPX4 expression and inhibited the anti-senescence effects of PL-Exos, which reduced p16^INK4A^ and p-p53 protein expression (Fig. 6G, H). Immunofluorescence staining of aging markers revealed a greater abundance of p21-stained TSPCs in the RSL3-treated group (Fig. 6I). In addition, Fig. 6J shows a notable increase in the proportion of SA-β-Gal-positive senescent TSPCs after treatment with RSL3. Furthermore, quantitative real-time PCR analysis revealed that the expression of the main senescence-associated secretory phenotype (SASP) markers, including MMP3, IL-6, IL-1β, and CXCL5, was greater in the RSL3 group than in the PL-Exos-only group (Fig. 6K). In summary, these findings suggested that PL-Exos might mitigate TSPC senescence by modulating AMPK/Nrf2/GPX4-dependent ferroptosis.

#### Platelet-derived exosomes promoted rotator cuff tendon-to-bone repair in vivo

**Fig. 7:**
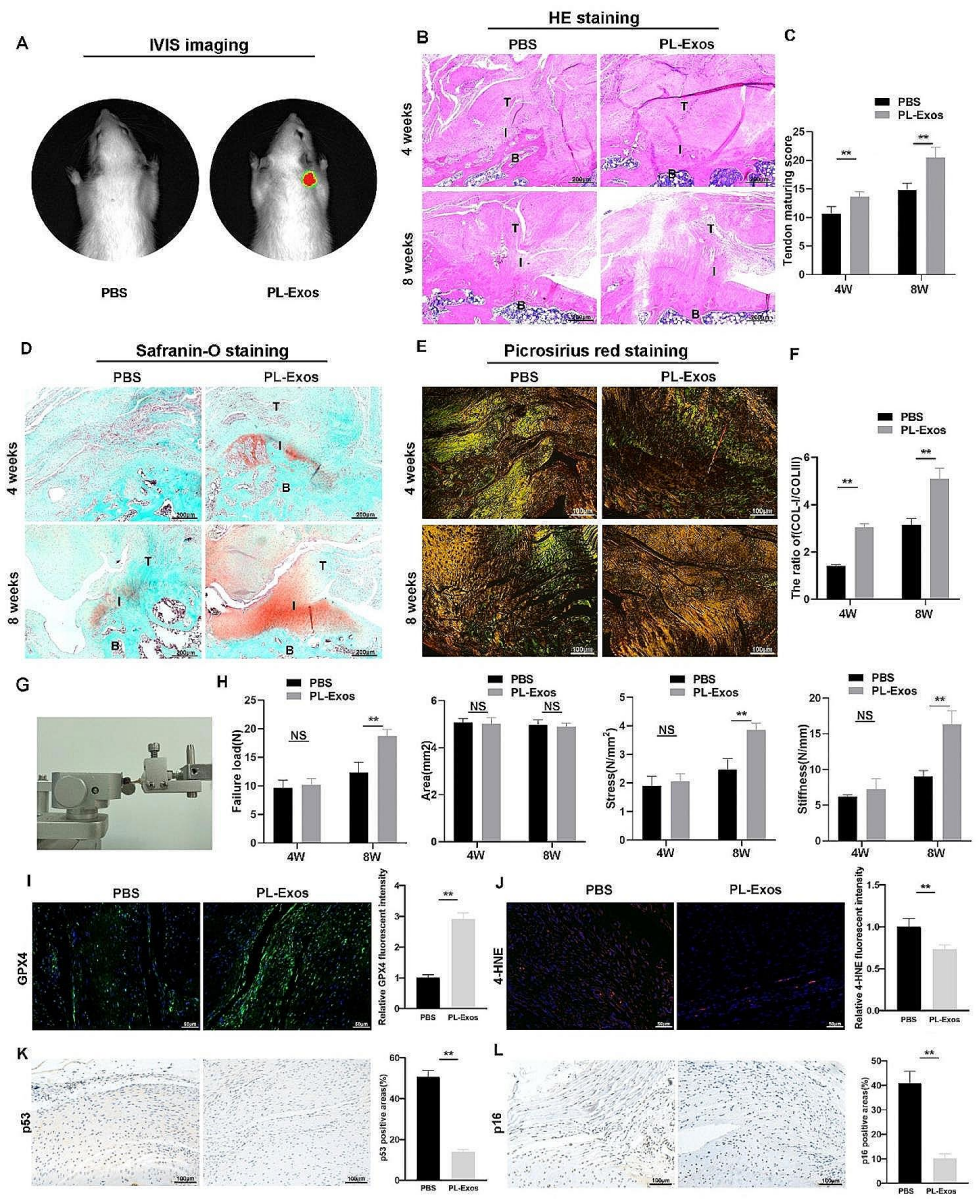
PL-Exos improved tendon-bone interface repair in vivo. (**A**) IVIS image of DIR-Exos in rats. (**B**) H&E staining images of the repaired tendon at 4 weeks and 8 weeks after the operation; B, bone; I, interface; T, tendon. (**C**) Quantitative analysis of tendon maturing scores; (**D**) Representative S&O staining image; (**E**) representative picrosirius red staining image; (**F**) quantitative analysis of COL-1/COL-3; (**H**,**I**) Biomechanical testing at 4 and 8 weeks postoperatively (ultimate load, stress, and stiffness, respectively); (**J**) Immunofluorescence image and quantitative analysis of GPX4 at 8 weeks after surgery; (**K**) Immunofluorescence image and quantitative analysis of 4-HNE; (**L**) Immunohistochemical assay images and quantitative analysis of p53; (**M**) Immunohistochemical assay images and quantitative analysis of p16^INK4A^; (F). ∗*p* < 0.05, ∗∗*p* < 0.01

PL-Exos were tracked in vivo after being labeled with DIR and injected into the shoulder joint after surgery. The fluorescent findings indicated that exosomes were present in the surgical shoulder joints (Fig. 7A), suggesting minimal or no transfer of exosomes. The accumulation in the joint could enhance the tendon healing process.

HE staining demonstrated a reduction in the infiltrating inflammatory cells in the PL-Exos group compared to the PBS group at 4 weeks. Disorganized fibroblastic ingrowth was observed in both groups. At 8 weeks, less infiltrating cells were observed in both groups. Moreover, the inflammation in the PL-Exos group decreased, resulting in a more compact extracellular matrix, increased alignment of fibers, and reduced cell count, resembling a healthy tendon composition. (Fig. 7B). Safranin-O staining revealed increased fibrocartilage formation at the tendon-to-bone interface in the PL-Exos group compared to the control group, which contributed to native enthesis formation (Fig. 7D). Figure 7C shows that the tendon maturation scores of the PL-Exos group were significantly greater than those of the control group at 4 and 8 weeks. When comparing the PL-Exos group to the PBS group, picrosirius red staining revealed that the former had a greater concentration of Type I fibers, which appeared yellow or red, and less type III collagen, which appeared green(Fig. 7E, F). Collagen fibers in the PL-Exos groups also showed a dense, well-organized structure that was disorganized and loose in the PBS group, suggesting that a well-aligned ECM structure might provide remodeling capabilities comparable to those of autogenous tendons.

To objectively determine the effects of the PL-Exos on the repaired tendon, we measured the mechanical characteristics of the tendon-bone attachment at four and eight weeks after the operation. Biomechanical testing revealed that there were no differences in ultimate load-to-failure, stress, or stiffness between the PBS group and PL-Exos group at the 4-week time point. However, the PL-Exos group had significant enhancements in ultimate load-to-failure, stress, and stiffness compared to the control group after 8 weeks (Fig. 7G-H). These findings showed that the healing capacity at the junction between the tendon and bone was enhanced in the PL-Exos group.

Furthermore, immunofluorescence staining revealed increased GPX4 expression and reduced 4-HNE expression in the PL-Exos group in comparison to the PBS group at 8 weeks (Fig. 4I-J). Immunohistochemical assays revealed a decrease in p16^INK4a^ and p53 expression in the PL-Exos group in comparison to the control group (Fig. 7K-L). These findings provided additional evidence that PL-Exos delayed tendon ferroptosis and aging.

## Discussion

Functional and prompt regeneration of tendons continues to be a significant challenge, particularly in older individuals. Platelet-rich plasma (PRP) and platelet lysate (PL) are types of platelet preparations that have shown significant growth in clinical and basic studies of tissue regeneration in recent years [16, 23, 24]. However, the main challenge to address with plasma products is the issue of allogenic immunogenicity, as well as the intricate composition and absence of standardized technical criteria [25, 26]. A recent study demonstrated that extracellular vesicles produced by platelets upon activation, also known as exosomes, are regarded as the effectors of PRP and PL [19]. Platelet-derived exosomes, a novel type of plasma products, are a cost-effective alternative to commercially available recombinant growth factors (GFs), which providing wider potential applications in the field of regenerative medicine.In this study, PL-Exos were successfully isolated from pure platelet lysate. Our cellular experiments have shown that PL-exosomes have a significant impact on promoting the proliferation of TSPCs and inhibiting cellular senescence when exposed to oxidative stress. Furthermore, we discovered that PL-Exos enhanced the processes of tenogenesis and fibrocartilage regeneration, as well as the biomechanical properties of the repaired tendon-bone enthesis in the rat model of RCT. These findings suggested that PL-Exos might be a promising strategy for the treatment of age-related tendon diseases.Tendon stem/progenitor cell (TSPC) senescence has been found to contribute to tendon aging and impaired tendon repair, resulting in age-related tendon disorders such as rotator cuff tears (RCTs). Traditionally, aging has been ascribed to the buildup of oxidative damage caused by reactive oxygen species (ROS) generated during cellular metabolic activities [27]. Ferroptosis is a type of regulated cell death that is distinguished by iron- and ROS-dependent lipid peroxidation. A recent study found that the Tfr1-iron axis is involved in muscle aging and regeneration through the activation of ferroptosis [28]. Increased accumulation of iron was observed in various aging tissues, leading to heightened oxidative stress caused by the production of highly toxic hydroxyl radicals through the Fenton reaction [29]. Notably, increased oxidative stress might facilitate the process of cellular senescence, sustaining a vicious cycle. The intricate relationship between ROS and the initiation of ferroptosis, as well as premature senescence, needs further research. In the present study, we observed a substantial decrease in the expression of glutathione (GSH) and glutathione peroxidase 4 (GPX4), along with elevated lipid peroxidation in both naturally aged and ROS-induced senescent TSPCs. Moreover, ACSL4, a positive ferroptosis-activating enzyme, was also activated in senescent TSPCs. Our study demonstrated that ferroptosis might participate in tendon degeneration and aging.

Platelet-derived exosomes have previously undergone extensive evaluation in the field of tissue regeneration [30, 31]. The regenerative effects of PL-Exos may be attributed to their ability to regulate inflammation and facilitate the proliferation and migration of stem cells [32, 33]. Recent studies also demonstrated that exosomes derived from plasma from young healthy humans prevent ferroptotic injury and aid in functional recovery following intracerebral hemorrhage [18]. However, few studies evaluating the effects of treatment with PL-Exos on cellular senescence and ferroptosis in tendons have been performed. In the present study, it was determined that PL-Exos might hinder oxidative stress-induced senescence. This was confirmed by the reduced expression of senescence-related proteins such as p16^INK4a^ and p-p53, as well as a decreased percentage of β-gal-positive cells during PL-Exos administration. Subsequently, we confirmed that PL-Exos facilitated the nuclear translocation of Nrf2 to upregulate GPX4 expression, attenuating lipid peroxidation and subsequent ferroptosis. Histological analysis and in vivo findings revealed that PL-Exos enhanced tendon-to-bone junction regeneration and improved mechanical functionality in a rotator cuff rupture rat model.

AMPK is a crucial kinase for maintaining energy balance and is involved in various physiological processes. The activation of AMPK due to energy stress was found to impede fatty acid synthesis by deactivating acetyl-CoA carboxylase (ACC), consequently hindering ferroptosis [11]. Additional research has demonstrated that the AMPK-mTOR pathway can induce ferroptosis through autophagy [34]. AMPK-mediated activation of Beclin1 promotes ferroptosis by inhibiting system X_c_^−^ activity [35]. Furthermore, AMPK phosphorylation facilitates the nuclear accumulation of Nrf2 [12, 36]. The transcription factor Nrf2 is widely recognized for its pivotal role in the process of antioxidation [37]. Activation of Nrf2 increases the expression of specific genes, including GPX4, and SLC7A11, which are recognized as crucial regulators of ferroptosis [8, 38, 39]. To further determine the underlying mechanisms, compound C and RSL3 were used. In the present study, PL-Exos promoted AMPK phosphorylation and facilitated the translocation of Nrf2 to the nucleus, thereby preventing ferroptosis. However, the effects of the PL-Exos were hindered by the intervention of the AMPK inhibitor Compound C. Furthermore, the inhibition of GPX4 by RSL3 was found to partially counteract the beneficial effects of the PL-Exos in terms of restoring the functional properties associated with senescence in TSPCs. These results suggested that PL-Exos exhibited antiaging effects on TSPCs by modulating the AMPK/Nrf2/GPX4 axis.

There are certain constraints in our study. Initially, we employed young TSPCs and aged TSPCs derived from both young and aged rat tendons. Clinically, the etiology of tendon aging and degeneration is multifactorial, involving factors such as aging, inflammation, and excessive mechanical loading. Further research is needed to investigate the precise mechanisms of tendon degeneration and aging in humans. In addition, the ideal frequency for exosome injection remains undetermined, and it is unclear whether multiple injections could yield superior outcomes compared to a single injection. Extensive study must be undertaken to confirm the correct dosage and injection frequency. Furthermore, given the wide range and intricate nature of the constituents found in PL-Exos, additional investigation is necessary to identify the precise compounds that enhance the healing of the tendons-bone interface in PL-Exos.

In summary, our findings demonstrated the crucial role of ferroptosis in the pathogenesis of TSPC aging and tendon regeneration. Furthermore, AMPK/Nrf2/GPX4 activation by PL-Exos inhibited ferroptosis, thereby suppressing the aging process. The in vivo findings demonstrated that PL-Exos facilitate tendon-to-bone regeneration in a rotator cuff tear model in rats. These data provide new insights into the unique pathogenic mechanism underlying TSPC senescence and ferroptosis and a new theoretical basis for the potential application of PL-Exos to restrain tendon degeneration and aging and promote tendon regeneration.

**Fig. 8:**
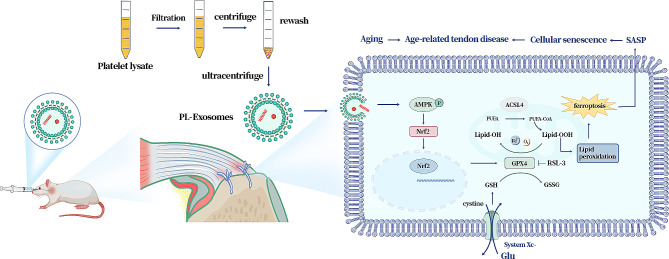
Schematic diagram of PL-Exos inhibiting TSPCs senescence and ferroptosis by regulating AMPK/Nrf2/GPX4 signaling and improve tendon-bone junction regeneration

## Data Availability

No datasets were generated or analysed during the current study.

## Abbreviations

AMPK: AMP-Activated Protein Kinase
TSPCs: Tendon Stem/Progenitor Cells
NRF2: Nuclear Factor erythroid 2-related factor 2
GPX4: Glutathione Peroxidase 4
PL-Exos: Platelet-Exosomes
PTGS2: Prostaglandin end peroxide synthase 2
SLC7A11: Solute Carrier family 7 member 11
CC: Compound C
RCT: Rotator Cuff Tears
